# Clinical characteristics and outcomes of Epstein-Barr virus viral load after allogeneic hematopoietic stem cell transplantation

**DOI:** 10.1007/s00277-023-05596-6

**Published:** 2023-12-29

**Authors:** Takafumi Tsushima, Shin-Ichi Masuda, Natsumi Yoda, Sayaka Kainuma, Chiharu Kimeda, Shiho Konno, Kazusuke Tanaka, Kosuke Matsuo, Sonoko Shimoji, Kenji Kimura, Hironori Arai, Yoshikazu Utsu, Ken-Ichi Imadome, Nobuyuki Aotsuka

**Affiliations:** 1 Department of Hematology and Oncology, Japanese Red Cross Narita Hospital, 90-1 Iida-Cho, Narita, 286-0041 Japan; 2 Department of Advanced Medicine for Virus Infections, National Center for Child Health and Development (NCCHD), Tokyo, Japan

**Keywords:** Epstein-Barr virus reactivation, Allogeneic hematopoietic stem cell transplantation, Rapid immunosuppressant dose reduction, High EBV viral load

## Abstract

Epstein-Barr virus (EBV) reactivation can occur following allogenic hematopoietic stem cell transplantation (allo-HSCT). However, the clinical characteristics and outcomes of EBV-viral load are not well known. Thus, we retrospectively analyzed the clinical features and prognostic impact of the EBV viral load in 121 allo-HSCT recipients from our hospital. EBV DNA quantification was performed in whole blood after transplantation. Patients were grouped based on whether EBV DNA quantification reached > 1000 copies/mL during follow-up (*N* = 50) or not (*N* = 71). Patients with EBV > 1000 EBV copies/mL were relatively more common in the groups with graft versus host disease (GVHD) prophylaxis including ATG, haploidentical donor type, peripheral blood as a donor source, and acute GVHD II–IV. The 20-month OS and DFS were not significantly different between patients with < 1000 EBV copies/mL and patients with > 1000 EBV copies/mL (20-month OS, 56.0% vs. 60.6%; *p* = 0.503, 20-month DFS, 50.0% vs. 57.7%; *p* = 0.179). Immunosuppressant (ISS) dose reduction was achieved after the maximum increase in EBV in 41/50 (82%) patients. Additionally, 30/50 (60%) patients achieved a 50% dose reduction or no restarting of ISS within 3 months of the maximum EBV increase. Among cases wherein EBV DNA quantification reached > 1000 copies/mL, those that achieved rapid dose reduction of ISS tended to have longer overall survival (“not reached” vs 5.4 months, *p* < 0.001) and disease-free survival (88.4 months vs 5.3 months, *p* < 0.001) than those in patients who did not. Our data highlight the importance of rapid ISS reduction in post-transplant EBV reactivation.

**Supplementary Information:**

The online version contains supplementary material available at 10.1007/s00277-023-05596-6.

## Introduction

Epstein-Barr virus (EBV) is the causative agent of infectious mononucleosis. EBV remains latent in lymphocytes after the initial infection and often reactivates after allogeneic hematopoietic stem cell transplantation (allo-HSCT) [1]. Occasionally, EBV reactivation progresses to lymphoproliferative disorder (LPD), and the types of LPD range from benign polyclonal B cell proliferation to malignant B/T cell lymphoma [2] [3]. LPD that occurs after allo-HSCT is of donor origin [4, 5].

The incidence of post-transplant lymphoproliferative disorders (PTLD) after allo-HSCT is approximately 1.0% [2]. The time of onset peaks between 60 and 90 days after HSCT, with 73% of cases occurring between 1 month and less than 6 months after HSCT and 9% between 6 months and up to 1 year. No cases occurred less than 1 month after HSCT, and cases rarely occurred after 1 year [2].

Risk factors for EBV reactivation include pre-transplant risk factors such as T cell depletion, EBV serology donor/recipient mismatch, cord blood transplantation, HLA mismatch, and second HSCT. Post-transplant risk factors include acute or chronic graft versus host disease (GVHD) requiring intensive immunosuppressant (ISS), high viral load, and mesenchymal stem cell therapy [6].

The European Conference on Infectious Diseases of Leukemia recommends weekly screening for EBV DNA in allo-HSCT recipients for at least 3 months after allo-HSCT [6].

A definitive diagnosis of LPD can be made by biopsy; however, this is difficult depending on the patient’s general condition after allo-HSCT. Monitoring EBV DNA in the peripheral blood is helpful in predicting the development of LPD [7]. The threshold value of EBV load can range from 1000 to over 40,000 copies/mL, depending on the sample, such as plasma, whole blood, and serum [8–13].

Due to the lack of direct treatment of EBV at the time of reactivation, prevention, ISS dose reduction, and preemptive treatment at the time of reactivation are important. The benefit of preemptive rituximab therapy is particularly notable in cases where EBV-infected cells are B cells. In contrast, rituximab can worsen the immunocompromised status after transplantation, thereby increasing the risk of infection and other complications. Reducing ISS dose is an important treatment for EBV reactivation; however, there are few established measures for ISS dose reduction, and the prognostic value of reduced ISS use has not been reported.

This study retrospectively analyzed the clinical features and prognostic impact of EBV viral load in allo-HSCT recipients.

## Methods

### Patient selection

This was a single-center, retrospective, observational study. A total of 121 consecutive patients who underwent allo-HSCT between December 2011 and February 2022 and EBV DNA quantification post-transplantation were included. Data on patients’ clinical findings were obtained from electronic medical records. The dataset was locked on February 28, 2022. Only cases in which the EBV viral load was measured over time after transplantation were included. This study was conducted in accordance with the Declaration of Helsinki 1964 and its later amendments or comparable ethical standards. This was approved by the Ethical Review Committee of the Japanese Red Cross Narita Hospital. This study was a retrospective observational study and obtaining written consent was not mandatory. All participants did not express any refusal to the study documents published on the Web using the opt-out method. The opt-out method was approved by our institutional review board.

### EBV viral load measurement and identification of EBV-infected cells

Antibody titers of EBV prior to transplantation were checked to confirm previous infections in all patients. EBV DNA was quantified in whole blood. The basic measurement frequency is once every 2 weeks or once a week until 100 days post-transplant, and once a month after 100 days. On the other hand, in cases with frequent visits or those receiving large amounts of immunosuppressants continuously due to GVHD, EBV was sometimes measured weekly, even after 100 days post-transplantation.

In some cases, EBV-infected cell identification analysis of peripheral blood was performed using multicolor flow cytometry (FCM) to test whether the infected cells were of B or T/NK cell origin. Identification of infected cells by FCM was performed at the discretion of the attending physician. Specifically, FCM is used to test for the loss of CD20 expression for the identification of EBV-infected cells. This was performed in some cases that did not respond well to rituximab.

### Background diseases and donor sources

Diseases that required and actually performed transplantation were included in the study. Malignant diseases included leukemia, myelodysplastic syndrome, myeloproliferative neoplasms, and malignant lymphoma, while benign diseases included aplastic anemia.

Patients who underwent allo-HSCT for chronic active EBV infection and EBV-HLH, wherein EBV initially infected T/NK cells, were excluded. Donor sources included bone marrow, peripheral blood stem cells, and cord blood (CB). There were no 8/8 HLA match in CB and all CB were counted as mismatched unrelated donor (MMUD) for donor type.

### Study definitions and endpoints

We grouped patients on whether EBV DNA levels reached > 1000 copies/mL in whole blood during follow-up (*N* = 50) or not (*N* = 71). Previous reports have used a cutoff of 1000 to over 40,000 copies/mL, which is an extensive range, and we used 1000 copies/mL as the cutoff to examine trends in a larger number of patients. We analyzed the relationship between the rate of ISS reduction and prognosis for the group that had > 1000 copies/mL. We also examined the ISS dose that could be reduced after the EBV viral load reached its maximum level. In particular, we examined whether existing ISS could be reduced to 50% of the dose within 3 months, considering the period when post-transplant EBV-LPD (PT-EBV-LPD) is most likely to occur late in the course of post-allo-HSCT. Even if the ISS dose could be reduced by 50% within 3 months, if the dose was subsequently increased after dose reduction, it was considered a case of immunosuppressive dose reduction failure. Although calcineurin inhibitors were the main immunosuppressive drugs, we also assessed whether the dosage of steroids and MMF could be reduced by 50% in 3 months. If the dosage was not reduced by 50%, it was considered as an ISS dose reduction failure. The decision to reduce the ISS dose was at the physician’s discretion and undertaken considering the EBV-viral load status, GVHD status, infection, and risk of disease recurrence. As a rule, ISS dose reductions were actively implemented when GVHD findings improved or disappeared, when severe viral infections occurred, or when signs of disease recurrence appeared. The use of rituximab was considered when the EBV viral load was particularly high, such as > 100,000 copies/mL, but it has also been used in cases of aggressive LPD like malignant lymphoma.

Disease-free survival (DFS) was calculated as the time interval from the date of transplantation to the date of first progression, recurrence, or death without progression. Overall survival (OS) was calculated as the time interval from the date of transplantation to the date of death from any cause.

### Statistical analysis

Baseline clinical characteristics were compared between the groups using the Mann–Whitney *U* test for continuous variables and Fisher’s exact test for categorical variables. Survival was analyzed using the Kaplan–Meier method and compared using the log-rank test to evaluate OS and DFS. Statistical significance was set at *p* < 0.05. Aplastic anemia was excluded from the prognosis stratified by complete remission (CR)/not CR. Since there are many cases of early death after transplantation, OS/DFS analysis by the landmark method at 3 months/6 months was also performed in some analysis. The prognostic impact was evaluated using univariate Cox proportional hazard analyses. The Scatter Plot was used for assessing the relationship between the two continuous variables. All statistical analyses were conducted using the EZR software (Saitama Medical Center, Jichi Medical University) [14].

## Results

### Clinical characteristics of patients based on maximum EBV-DNA after allo-HSCT

The clinical characteristics of patients based on the presence of a high EBV viral load (> 1000 EBV copies/mL) are summarized in Table 1. The median age of all patients was 53.0 years, and 64.5% were male. EBV-DNA in 50 patients (41.3%) was > 1000 EBV copies/mL, while 71 (58.6%) did not. The underlying diseases were acute myeloid leukemia/myelodysplastic syndrome (68.6%), chronic myeloid leukemia (2.5%), myeloproliferative neoplasms (3.3%), acute lymphoblastic leukemia (16.5%), non-Hodgkin lymphoma/Hodgkin lymphoma (6.6%), and aplastic anemia (2.5%). Complete remission (CR) was achieved in 51.2% of the patients at the time of transplantation. Among patients with > 1000 EBV copies/mL, 48% achieved CR at the time of transplantation, compared to 53.5% in patients with < 1000 EBV copies/mL. There were no differences in conditioning, Eastern Cooperative Oncology Group (ECOG)- performance status (PS), chronic GVHD, or cytomegalovirus activation after transplantation between the patients with > 1000 EBV copies/mL and patients with < 1000 EBV copies/mL. In contrast, patients with > 1000 EBV copies/mL had a higher frequency of anti-thymocyte globulin (ATG) haplo-transplantation, acute GVHD (grade 2 or higher), and peripheral blood stem cells as the graft source.

**Table 1:** Clinical characteristics of patients based on whether maximum EBV-DNA was > 1000 EBV copies/mL after allo-HSCT

Characteristics	Total (*n* = 121)	Patients with > 1000 EBV copies/mL (*n* = 50)	Patients with < 1000 EBV copies/mL (*n* = 71)	*p*-value
Age at transplant, years, median (range)	53 (21–70)	55 (25–69)	51 (21–70)	0.214
Male sex, *N* (%)	78 (64.5)	30 (60.0)	48 (67.6)	0.443
Underlying disease, *N* (%)				0.166
AML/MDS	83 (68.6)	36 (72.0)	47 (66.2)	
CML	3 (2.5)	3 (6.0)	0 (0.0)	
MPN	4 (3.3)	2 (4.0)	2 (2.8)	
ALL	20 (16.5)	7 (14.0)	13 (18.3)	
NHL/HL	8 (6.6)	1 (2.0)	7 (9.9)	
AA	3 (2.5)	1 (2.0)	2 (2.8)	
Disease status at transplantation, *N* (%)				0.817
In any CR	62 (51.2)	24 (48.0)	38 (53.5)	
Not CR	56 (46.3)	25 (50.0)	31 (43.7)	
Other	3 (2.5)	1 (2.0)	2 (2.8)	
Conditioning, *N* (%)				0.848
MAC	42 (34.7)	18 (36.0)	24 (33.8)	
RIC	79 (65.3)	32 (64.0)	47 (66.2)	
ECOG PS, *N* (> 0, %)	51 (42.1)	23 (46.0)	28 (39.4)	0.575
CMV reactivation after transplantation, *N* (%)	33 (27.3)	19 (26.8)	14 (28.0)	1.000
Prophylaxis of GVHD, *N* (%)				< 0.001
ATG + mPSL + CNI	34 (28.1)	26 (52.0)	8 (11.3)	
PTCY + TAC + MMF	6 (5.0)	2 (4.0)	4 (5.6)	
Short MTX + CNI	69 (57.0)	20 (40.0)	49 (69.0)	
MMF + CNI	12 (9.9)	2 (4.0)	10 (14.1)	
Graft source, *N* (%)				0.001
PB	52 (43.0)	29 (58.0)	23 (32.4)	
BM	31 (25.6)	14 (28.0)	17 (23.9)	
CB	38 (31.4)	7 (14.0)	31 (43.7)	
Donor type, *N*				< 0.001
Haploidentical	39 (32.2)	27 (54.0)	12 (16.9)	
Matched related donor	13 (10.7)	1 (2.0)	12 (16.9)	
Matched unrelated donor	24 (19.8)	12 (24.0)	12 (16.9)	
Mismatched unrelated donor	45 (37.2)	10 (20.0)	35 (49.3)	
Acute GVHD grades II-IV, *N* (%)	61 (50.4)	31 (62.0)	30 (42.3)	0.042
Chronic GVHD, *N* (%)				0.562
Mild	33 (27.3)	15 (30.0)	18 (25.4)	
Moderate	19 (15.7)	9 (18.0)	10 (14.1)	
Severe	3 (2.5)	2 (4.0)	1 (1.4)	
Re-transplantation, *N* (> 0, %)	21 (17.4)	9 (18.0)	12 (16.9)	1.000

Abbreviations: *AA*, aplastic anemia; *ALL*, acute lymphoblastic leukemia; *allo-HSCT*, allogeneic-hematopoietic stem cell transplantation; *AML*, acute myeloid leukemia; *ATG*, antithymocyte globulin; *BM*, bone marrow; *CB*, cord blood; *CML*, chronic myeloid leukemia; *CMV*, cytomegalovirus; *CNI*, calcineurin inhibitor; *CR*, complete remission; *EBV*, Epstein-Barr virus; *ECOG*, Eastern Cooperative Oncology Group; *GVHD*, graft versus host disease; *HL*, Hodgkin lymphoma; *MAC*, myeloablative conditioning; *MDS*, myelodysplastic syndromes; *MMF*, mycophenolate mofetil; *MPN*, myeloproliferative neoplasms; *mPSL*, methylprednisolone; *MTX*, methotrexate; *NHL*, non-Hodgkin lymphoma; *PB*, peripheral blood; *PS*, performance status; *PTCY*, post-cyclophosphamide; *RIC*, reduced intensity conditioning; *TAC*, tacrolimus

### *Clinical characteristics of patients with* > *1000 EBV copies/mL*

We then performed an additional analysis of the 50 patients with a high EBV viral load (> 1000 copies/mL) after transplantation (Table 2). Sixty percent of the patients reached the maximum EBV DNA level before post-transplant day 100, while 38% reached the maximum level between days 100 and 1000, and 2% reached maximum level after day 1000. The viral load was 3log (> 1000 copies/mL) in 52%, 4log (> 10,000 copies/mL) in 32%, 5log (> 100,000 copies/mL) in 8%, and 6log (> 1,000,000 copies/mL) in 8% of the patients. PT-EBV-LPD was diagnosed using lymph node biopsy or based on new multiple lymphadenopathies on CT in 10% of cases. The median viral load in patients with LPD was 1,500,000 EBV copies/mL (range 76,000–4,900,000 copies/mL). Tacrolimus and cyclosporine were used in 28% and 56% of the cases, respectively, when EBV reached the maximum viral load. Following maximum EBV increase, rituximab was used in six cases (12%), and 30 cases (60%) showed ISS dose reduction to 50% within 3 months. The percentage of patients with lymphocytes at least 1000/µL at maximum detection was 22/50 (44.0%). No correlation was observed between lymphocyte counts and maximum EBV-DNA levels, with a Spearman’s rank correlation coefficient − 0.066 (Online Resource.1).

**Table 2:** Clinical characteristics of patients with > 1000 EBV copies/mL (*N* = 50)

Characteristics	Total (*N* = 50)
Days after transplantation at max EBV increase, *N* (%)	
Days < 100	30 (60.0)
100 < days < 365	11 (22.0)
365 < days < 1000	8 (16.0)
1000 < days	1 (2.0)
Viral load at max EBV increase, *N* (%)	
> 1000 copies/mL	26 (52.0)
> 10,000 copies/mL	16 (32.0)
> 100,000 copies/mL	4 (8.0)
> 1,000,000 copies/mL	4 (8.0)
Peripheral blood lymphocyte count at max EBV increase, cells/μL, median (range)	740 (19–7504)
Occurrence of LPD, *N* (%)	5 (10.0)
The use of ISS at max EBV increase, *N* (%)^‡^	
TAC	14 (28.0)
CyA	28 (56.0)
PSL	24 (48.0)
Others	1 (2.0)
ISS reduction after max EBV increase, *N* (%)	41 (82.0)
Achieving 50% dose reduction or no restarting of ISS within 3 months after max EBV increase, *N* (%)	30 (60.0)
1 or 2 log decrease in viral load after ISS reduction, *N* (%)	37 (74.0)
The use of rituximab against EBV increase, *N* (%)	6 (12.0)

^**‡**^Some cases used both PSL and CNI; hence, the total numbers do not match

Abbreviations: *CyA*, cyclosporin; *EBV*, Epstein-Barr virus; *ISS*, immunosuppressant; *LPD*, lymphoproliferative disorder; *PSL*, prednisolone; *TAC*, tacrolimus

### Survival of allo-HSCT patients with EBV and ISS reduction

The median follow-up period was 16.2 (1.17–132.17/SD 34.6) months. EBV-PTLD began to appear as early as 3–6 months after transplantation in adults [15], and the previous report showed 3-year OS 47.3% for EBV-LPD after transplantation [16]; thus, we set the OS time point at approximately 20 months, which is close to the median observation period.

**Fig. 1:**
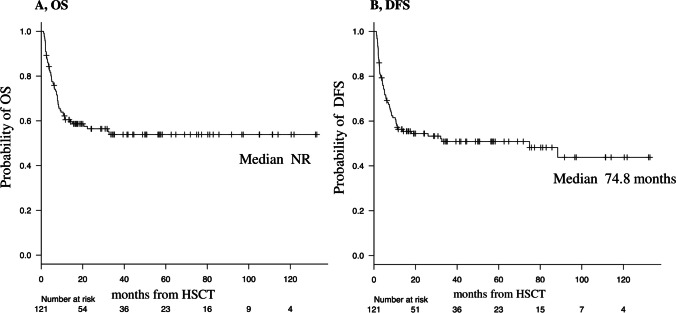
OS and DFS in all patients. DFS, disease-free survival; OS, overall survival

**Fig. 2:**
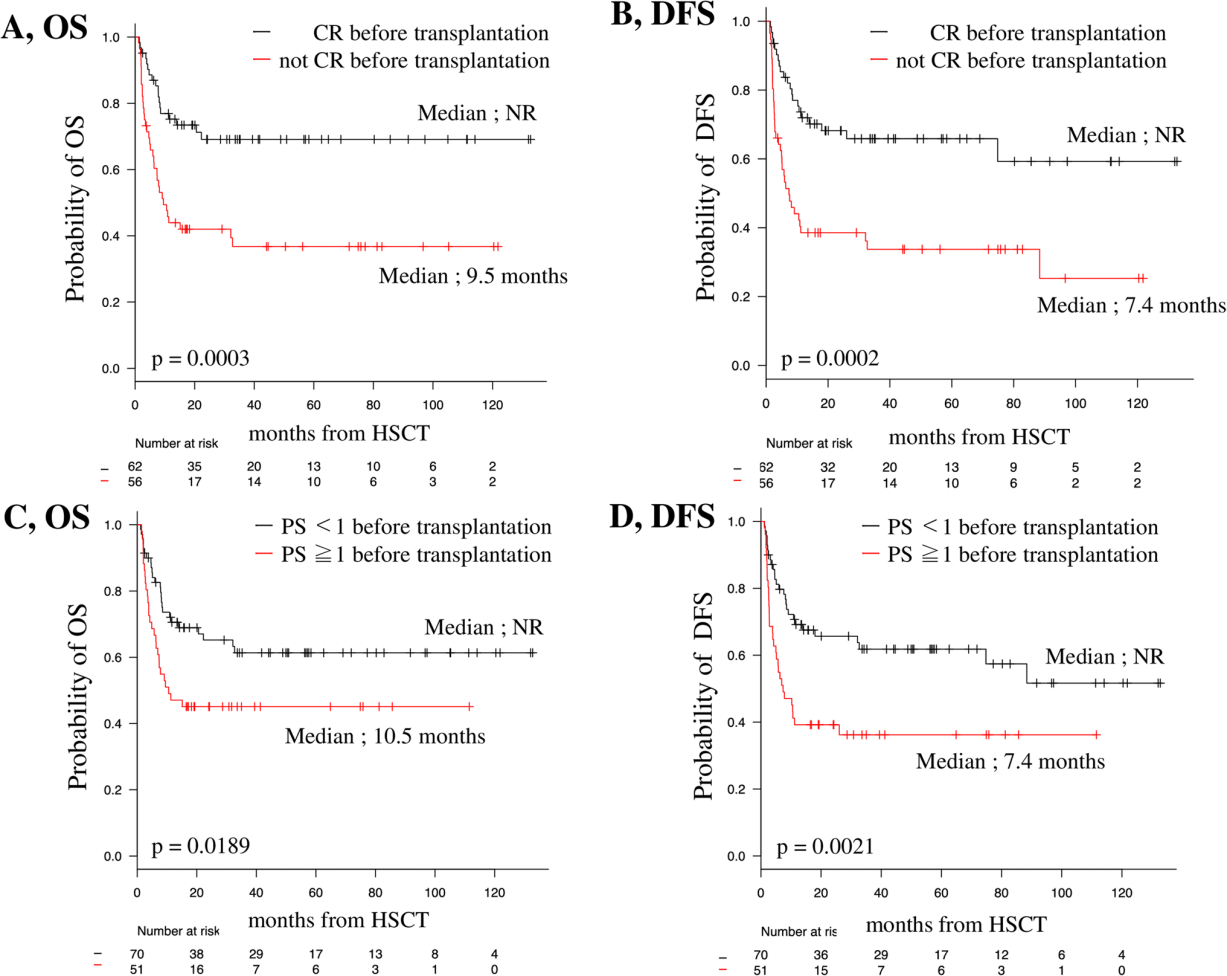
OS and DFS based on the presence of CR and PS before transplantation in all patients. CR, complete remission; DFS, disease-free survival; OS, overall survival; PS, performance status

The median and 20-month OS rate of all patients were “not reached” and 58.7% (95% confidence interval [CI], 49.3–67.0%), respectively (Fig. 1A). The median and 20-month DFS rate of all patients were 74.8 months and 54.6% (95% CI, 45.1–63.0%), respectively (Fig. 1B). Patients who achieved CR before allo-HSCT showed significantly longer OS and DFS than those in patients who did not (20-month OS, 73.5% vs. 42.0% [95% CI, 60.4–82.9 vs. 28.9–54.6]; *p* = 0.0003; Fig. 2A; 20-month DFS, 68.3% vs. 38.5% [95% CI, 54.8–78.5 vs. 25.8–51.1]; *p* = 0.0002; Fig. 2B).

Patients with good PS (PS < 1) before allo-HSCT showed significantly longer OS and DFS than patients with poor PS (PS ≧ 1) before allo-HSCT (20-month OS, 69.0% vs. 45.1% [95% CI, 56.5–78.6 vs. 31.2–58.0]; *p* = 0.0189; Fig. 2C; and 20-month DFS, 65.8% vs. 39.2% [95% CI, 53.1–75.9 vs. 26.0–52.2]; *p* = 0.0021; Fig. 2D).

**Fig. 3:**
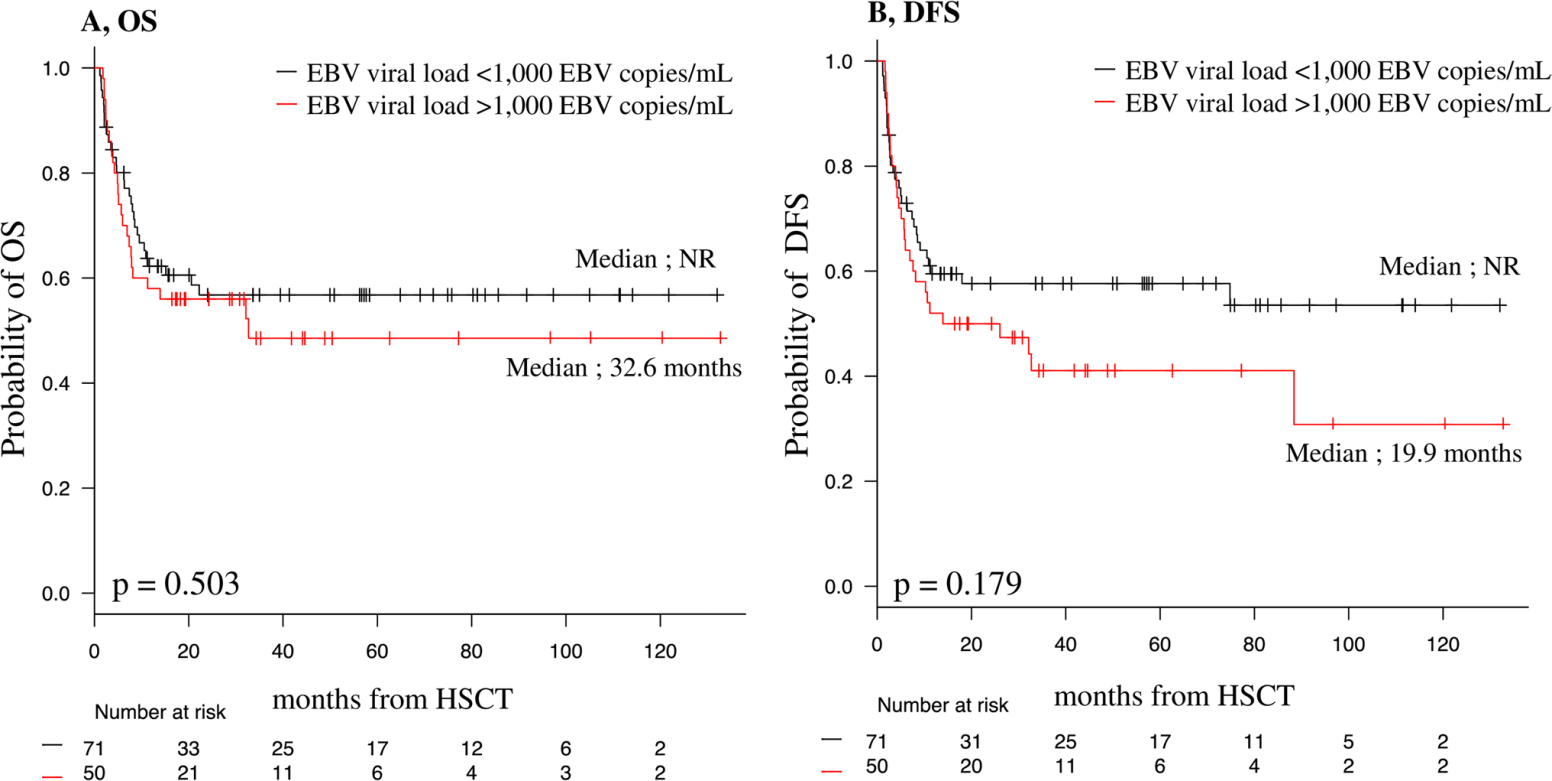
OS and DFS based on the presence of a high EBV viral load (> 1000 copies/mL) after transplantation. DFS, disease-free survival; EBV, Epstein-Barr virus; OS, overall survival

The 20-month OS (Fig. 3A) and DFS (Fig. 3B) were not significantly different between patients with < 1000 EBV copies/mL and patients with > 1000 EBV copies/mL (20-month OS, 56.0% vs. 60.6% [95% CI, 41.2–68.4% vs. 48.0–71.1%]; *p* = 0.503; 20-month DFS, 50.0% vs. 57.7% [95% CI, 35.6–62.8% vs. 45.0–68.4%]; *p* = 0.179).

Because patients could have died or showed progression/relapse before reaching their highest EBV viral load, a landmark OS analysis was performed at 3/6 months in the EBV viral load analysis. Patients with > 1000 EBV copies/mL were not significantly different in OS or DFS compared to patients with < 1000 EBV copies/mL (3-month landmark/20-month OS, 63.6% vs. 70.4%, *p* = 0.291, 3-month landmark/20-month DFS, 61.0% vs. 71.8%, *p* = 0.051, and 6-month landmark/20-month OS, 80.0% vs. 75.5%, *p* = 0.849, 6-month landmark/20-month DFS, 78.1% vs. 78.9%, *p* = 0.273) (Online Resource.2).

**Fig. 4:**
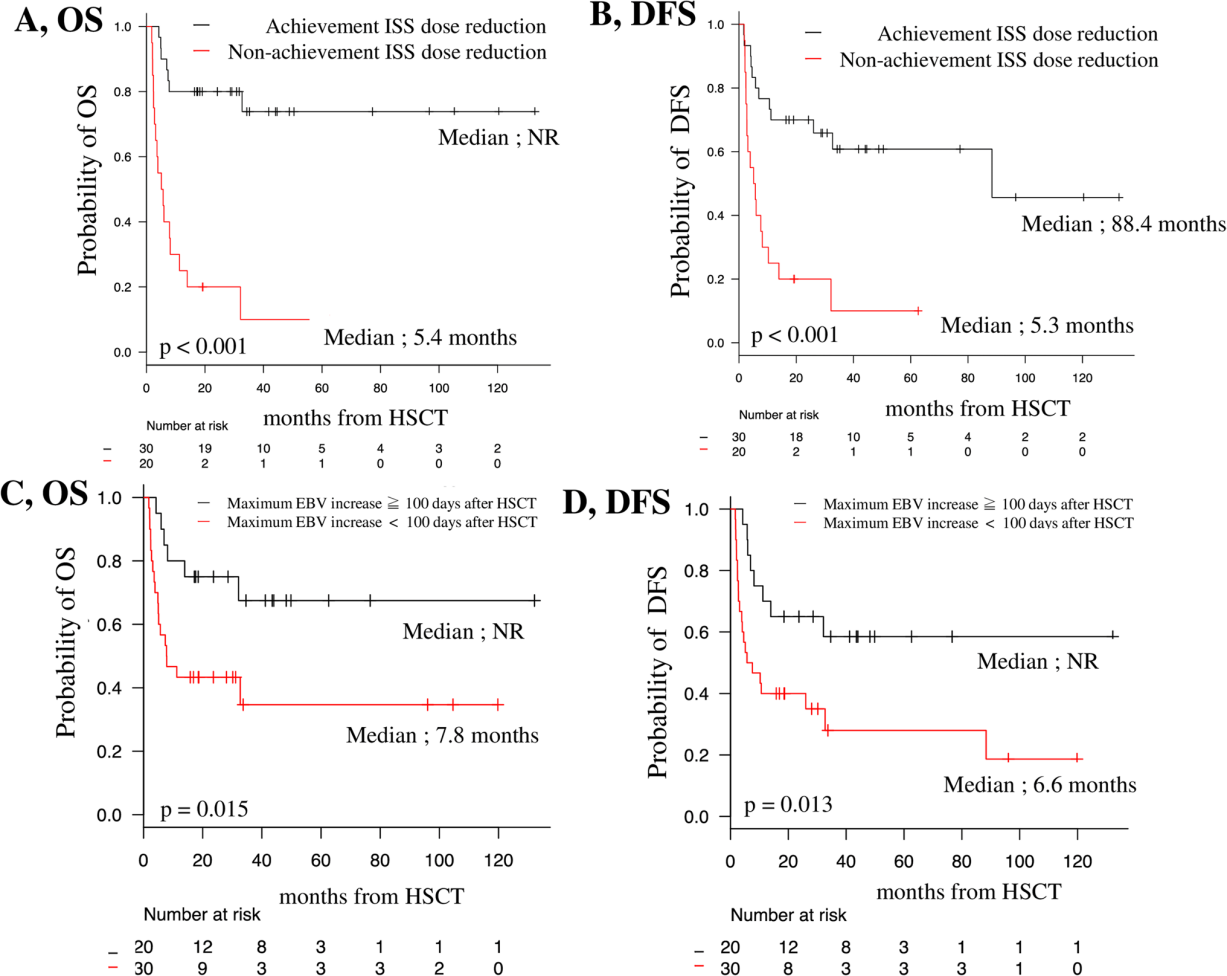
**A** OS and **B** DFS of patients with > 1000 EBV copies/mL based on the achievement of 50% dose reduction or no restarting of ISS within 3 months after the maximum EBV increase. **C** OS and **D** DFS based on the presence of a maximum EBV increase < 100 days after HSCT in patients with > 1000 EBV copies/mL. DFS, disease-free survival; EBV, Epstein-Barr virus; HSCT, hematopoietic stem cell transplantation; ISS, immunosuppressants; OS, overall survival

Further focusing on patients with > 1000 EBV copies/mL, those who achieved 50% ISS dose reduction, including not restarting ISS, within 3 months after HSCT did not have a significant difference in the frequency of chronic GVHD (10.0% vs. 15.0%; *p* = 0.672) or rituximab use (56.7% vs. 45.0%; *p* = 0.565) than those who did not achieve 50% dose reduction. However, these patients showed significantly longer OS and DFS than those who did not achieve 50% dose reduction (20-month OS, 80.0% vs. 20.0% [95% CI, 60.8–90.5 vs. 6.2–39.3]; *p* < 0.0001; Fig. 4A; and 20-month DFS, 70.0% vs. 20.0% [95% CI, 50.3–83.1 vs. 6.2–39.3]; *p* < 0.0001; Fig. 4B).

In considering dose reduction of ISS, an additional landmark analysis was performed because there were cases of death/progression before ISS dose reduction. Additional analysis focused only on patients who developed EBV reactivation, particularly within 12 months of transplantation. Those who achieved 50% ISS dose reduction showed significantly longer OS and DFS than those who did not achieve 50% dose reduction (3-month landmark/median OS, NR vs. 6.9 months, *p* = 0.001, 3-month landmark/median DFS, 88.4 months vs. 7.6 months, *p* = 0.007, and 6-month landmark/median OS, NR vs. 12.6 months, *p* = 0.025, 6-month landmark median DFS, 88.4 months vs. 12.1 months, *p* = 0.018) (Online Resource. 3).

Rituximab was used in 10% (3/30) of patients in the group that achieved rapid ISS reduction and in 15% (3/20) in the group that did not. There were two deaths due to EBV-LPD, and a rapid ISS dose reduction could not be achieved; however, rituximab was administered. Patients who achieved a 50% ISS dose reduction, including not restarting ISS, within 3 months, were able to achieve a 1 or 2log reduction in EBV DNA levels than those who did not achieve 50% dose reduction (86.7% vs. 55.0%; *p* = 0.021).

Patients who reached the maximum EBV-DNA level over 100 days post-transplantation had better OS and DFS than those who reached the maximum EBV DNA level within 100 days post-transplantation (20-month OS, 75.0% vs. 43.3% [95% CI, 50.0–88.7 vs. 25.6–59.9]; *p* = 0.015; Fig. 4C; and 20-month DFS, 73.5% vs. 43.3% [95% CI, 47.5–88.1 vs. 25.6–59.9]; *p* = 0.013; Fig. 4D).

### Univariate Cox regression analysis of OS and DFS

Univariate analysis was performed to identify factors independently associated with survival in patients with > 1000 EBV copies/mL after transplantation (Table 3). In the univariate analysis, achieving 50% dose reduction or not restarting ISS within 3 months after the maximum EBV increase, PS, and disease status were significantly associated with OS. ATG use, achieving 50% dose reduction or not restarting ISS within 3 months after the maximum EBV increase, and PS and disease status were significantly associated with DFS.

**Table 3:** Univariate Cox regression analysis of OS and DFS in patients with > 1000 EBV copies/mL

	Variables	Univariate analysis HR (95% CI)	*p*-value
OS	Use of ATG (prophylaxis of GVHD)	1.73 (0.97–2.99)	0.063
	PTCY-Haplo	1.51 (0.54–4.18)	0.432
	Achieving 50% dose reduction or no restarting of ISS within 3 months after max EBV increase	0.14 (0.06–0.34)	< 0.001
	ECOG PS, *N* (> 0, %)	1.89 (1.10–3.25)	0.021
	Disease status at transplantation (not CR)	2.74 (1.55–4.87)	< 0.001
	Acute GVHD grades II-IV	1.27 (0.74–2.18)	0.382
DFS	Use of ATG (prophylaxis of GVHD)	2.23 (1.31–3.79)	< 0.001
	PTCY-Haplo	1.37 (0.49–3.79)	0.541
	Achieving 50% dose reduction or no restarting of ISS within 3 months after max EBV increase	0.23 (0.10–0.51)	< 0.001
	ECOG PS, *N* (> 0, %)	2.20 (1.31–3.69)	0.002
	Disease status at transplantation (not CR)	2.68 (1.56–4.59)	< 0.001
	Acute GVHD grades II-IV	1.31 (0.79–2.20)	0.298

Abbreviations: *ATG*, antithymocyte globulin; *CI*, confidence interval; *CR*, complete remission; *DFS*, disease-free survival; *EBV*, Epstein-Barr virus; *ECOG*, Eastern Cooperative Oncology Group; *GVHD*, graft versus host disease; *HR*, hazard ratio; *ISS*, immunosuppressant; *OS*, overall survival; *PS*, performance status; *PTCY*, post-cyclophosphamide

### Characteristics of cases with high EBV viral load despite the end of ISS agents

In five cases, the amount of EBV DNA in the peripheral blood continued to increase even after ISS ended (Table 4). The age at transplantation ranged from 47 to 66 years, and the background diseases included acute lymphoblastic leukemia (one case), myeloproliferative neoplasms (one case), non-Hodgkin lymphoma (one case), and myelodysplastic syndrome (two cases). Conditioning included myeloablative conditioning in two cases and reduced intensity conditioning in three cases. GVHD prophylaxis included ATG in only one case, and no post-transplant cyclophosphamide haplo-transplantation was done. None of the patients had post-transplant relapse or progression to PT-EBV-LPD. All patients were followed up without rituximab. In all cases, EBV did not disappear, and a high viral load (> 10,000 copies/mL) was maintained.

**Table 4:** Characteristics of patients with > 1000 EBV copies/mL despite the end of immunosuppression agents

Patient	Age	Sex	Underling disease	Conditioning	GVHD prophylaxis	Disease status at SCT	Relapse	LPD	Day when ISS were stopped	Days after SCT at max EBV increase	PB lymphocyte count at max EBV increase
1	66	Male	NHL	RIC	MMF + CNI	Not CR	None	None	Day 91	Day 420	4346 cells/μL
2	57	Male	MPN	MAC	MTX + CNI	CR	None	None	Day 106	Day 266	3168 cells/μL
3	47	Female	ALL	MAC	ATG + mPSL + CNI	Not CR	None	None	Day 322	Day 469	1360 cells/μL
4	59	Female	MDS	RIC	MTX + CNI	CR	None	None	Day 709	Day 1143	1408 cells/μL
5	66	Male	MDS	RIC	MTX + CNI	CR	None	None	Day 218	Day 976	2128 cells/μL

Abbreviations: *ALL*, acute lymphoblastic leukemia; *ATG*, antithymocyte globulin; *CNI*, calcineurin inhibitor; *CR*, complete remission; *EBV*, Epstein-Barr virus; *GVHD*, graft versus host disease; *ISS*, immunosuppressant; *LPD*, lymphoproliferative disorder; *MAC*, myeloablative conditioning; *MDS*, myelodysplastic syndromes; *MMF*, mycophenolate mofetil; *MPN*, myeloproliferative neoplasms; *mPSL*, methylprednisolone; *MTX*, methotrexate; *NHL*, non-Hodgkin lymphoma; *PB*, peripheral blood; *RIC*, reduced intensity conditioning; *SCT*, stem cell transplantation

## Discussion

Allogeneic PT-EBV-LPD treatment includes rituximab treatment, reduction of ISS, donor lymphocyte infusion, and chemotherapy [6, 7, 15]. The benefits of rituximab are especially marked [16]. Since it is possible that LPD of T/NK cell types may be present, identification of EBV-infected cells using flow cytometry should also be undertaken [17].

The use of rituximab is an important component in the treatment of PT-EBV-LPD. Depending on the malignancy grade or CD20 expression in PTLD, there have been cases in which rituximab alone was not curative, including B cell LPD [18].

In our study, rituximab was used in some patients with high EBV viral loads, and CD20 expression decreased rapidly during rituximab treatment. The mechanism for this is known to involve the downregulation of surface CD20 [18]. In such cases, a combination of other chemotherapies, such as the CHOP regimen, may be effective. The efficacy of rituximab is more limited for EBV-LPDs localized in the central nervous system because of the blood–brain barrier more than other regions. Hence, treatment strategies other than rituximab should be considered [19]. A special approach involving intrathecal rituximab has been reported for the central nervous system PT-EBV-LPD. However, this approach has not been widely established [20].

A few established indicators for the specific rate of ISS dose reduction should be used in post-transplant EBV increase. In fact, there is only a small amount of literature examining specific methods of ISS reduction after EBV reactivation. Cesaro et al. reported a sustained decrease of at least 20% of the daily dose of ISS drugs for PT-EBV-LPD, except for low-dose corticosteroid therapy [21]. Hematopoiesis is sometimes unstable after transplantation, and chemotherapy for EBV is more difficult due to the decrease in hematopoiesis. Therefore, we believe that our strategy of ISS dose reduction by 50% within 3 months after EBV increase may be beneficial. Prevention and treatment of GVHD are key to the success of this strategy. Nevertheless, rituximab is also effective in the polyclonal phase, and a combination of rituximab therapy and the rapid reduction of ISS should be combined when EBV viral load is elevated.

In our study, the EBV viral load was not a prognostic factor. However, it has been suggested that rapidly reducing ISS dose may prevent PT-EBV-LPD. A reduction in EBV DNA levels was also predominant in patients who achieved rapid ISS dose reductions. Moreover, it is expected that chronic GVHD would not be as frequent in patients whose ISS were reduced to 50% within 3 months after increasing EBV; however, the frequency of chronic GVHD was not significantly different between the groups that had rapidly reduced ISS dose and those that did not. In cases with GVHD, it may be important to reduce ISS use according to the grade of GVHD; however, in cases without GVHD, a more aggressive and rapid ISS reduction may be helpful in controlling the current disease and preventing EBV reactivation. In our study, the prognosis may be worse in cases of an early increase in EBV after transplantation.

In our analysis, there were several cases of persistently high EBV viral load after allo-HSCT, despite the absence of ISS. The development of EBV-positive Hodgkin lymphoma has been previously reported in cases wherein CD4-positive lymphocytes in the peripheral blood recovered after HIV treatment [22]. These are cases of EBV-positive lymphoma in patients whose immune competence has recovered, and a different mechanism must be assumed for immunocompromised methotrexate-associated lymphproliferative disorder or other iatrogenic immunodeficiency-associated LPD. The recovery of CD4-positive T cells is expected to result in the increased production of various cytokines, wherein some of these T cell cytokines may act on the proliferation of the B cell lineage [23]. In our cases with some CD4 monitoring, there were some cases wherein recovery of CD4-positive lymphocytes was observed. Recovery of CD4-positive lymphocytes may be a factor in the cases with persistently high EBV viral load observed after completion of ISS. Surprisingly, these cases were uneventful even in patients who did not receive rituximab or other therapeutic chemotherapy.

The cohort in our study was a sample of patients for whom EBV could be measured over time after allo-HSCT—long-term survivors. This allowed for a relatively long observation period, and OS tended to be slightly better than prognosis of the typical allo-HSCT patient. However, the survival of this cohort has significant differences based on the presence of CR and good or bad PS and is not considered a cohort that deviates from the typical allo-HSCT patient.

CB and ATG-Haplo transplants are both risk factors for EBV reactivation. ATG-Haplo transplantation has been reported as a risk factor for increased EBV [24].

The relatively low proportion of patients who underwent CBT in the patients with > 1000 EBV copies/mL group may be attributed to the disproportionately high frequency of ATG-Haplo transplants in this group.

The limitations of this study include its small sample size and retrospective nature. The treatment was heterogeneous because of the long study period and the presence of various diseases. EBV DNA quantification in peripheral blood is not always helpful due to exceptions wherein PT-EBV-LPD develops without obvious EBV viremia and with low levels of EBV DNA in the blood [25]. Despite these limitations, to the best of our knowledge, this is the first study to describe a reduction in EBV reactivation and improved prognosis by rapid dose reduction of ISS after allo-HSCT.

In conclusion, we identified that after detecting a high EBV viral load, OS and DFS were significantly lower in the group of patients who could not reduce ISS use by 50% or less within 3 months than that in the group of patients who could. Although there have been several cases of long-term persistence of a high EBV viral load despite the end of ISS therapy, these cases were uneventful. To confirm our results, an independent study with larger and more detailed investigations with a prospective assessment is required.

### Supplementary Information

Below is the link to the electronic supplementary material.

Supplementary file1 (TIFF 1034 KB)

Supplementary file2 (TIFF 1376 KB)

Supplementary file3 (TIFF 1378 KB)
