# Supplementary figures and images for: Clinical characteristics and outcomes of Epstein-Barr virus viral load after allogeneic hematopoietic stem cell transplantation
Source: Ann Hematol. 2023 Dec 29;103(3):935–46. doi: 10.1007/s00277-023-05596-6 (PMC10867052; doi:10.1007/s00277-023-05596-6)

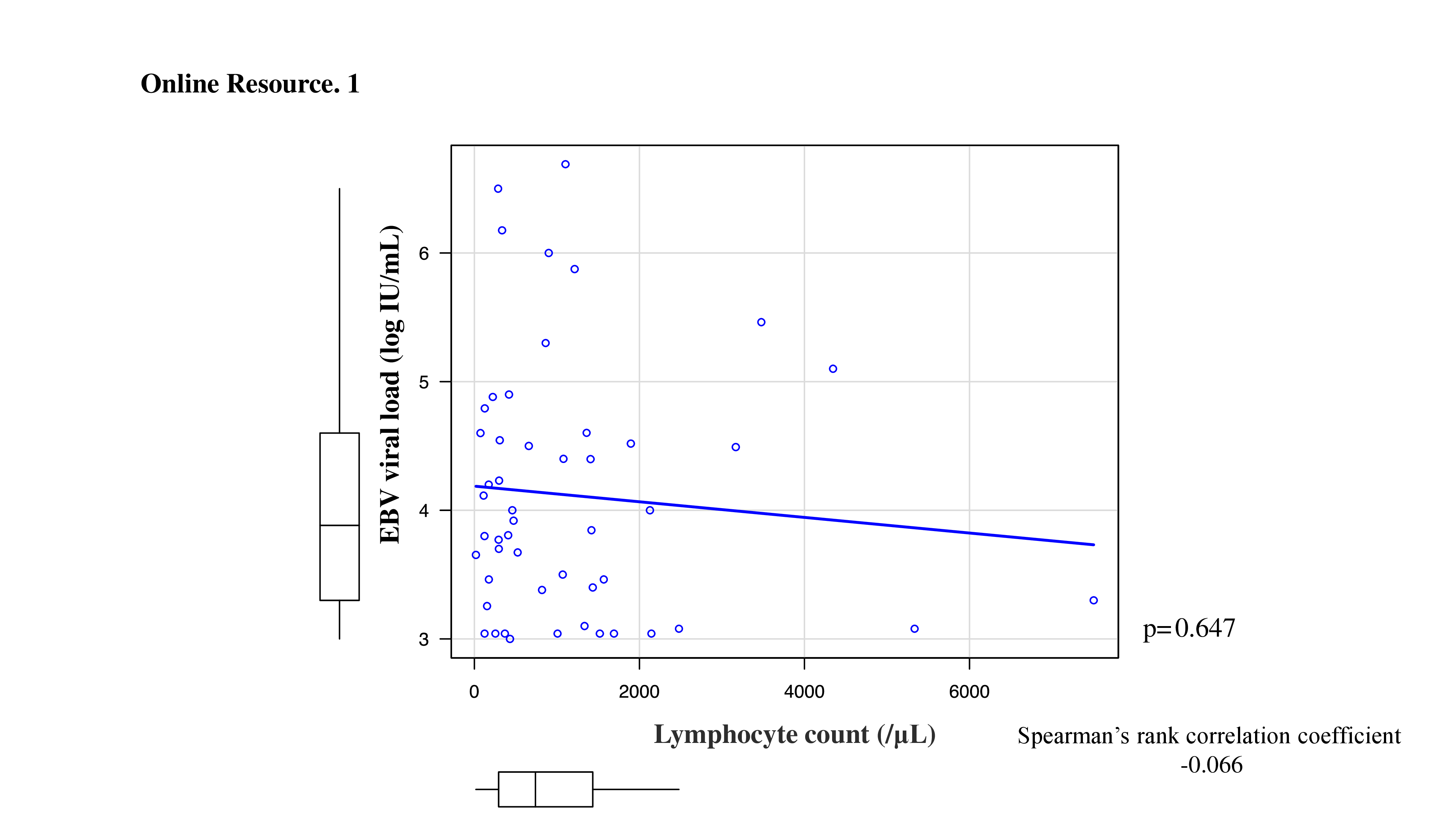

Supplement: Supplementary file 1 — Supplementary file1 (TIFF 1034 KB) [file 277_2023_5596_MOESM1_ESM.tiff]

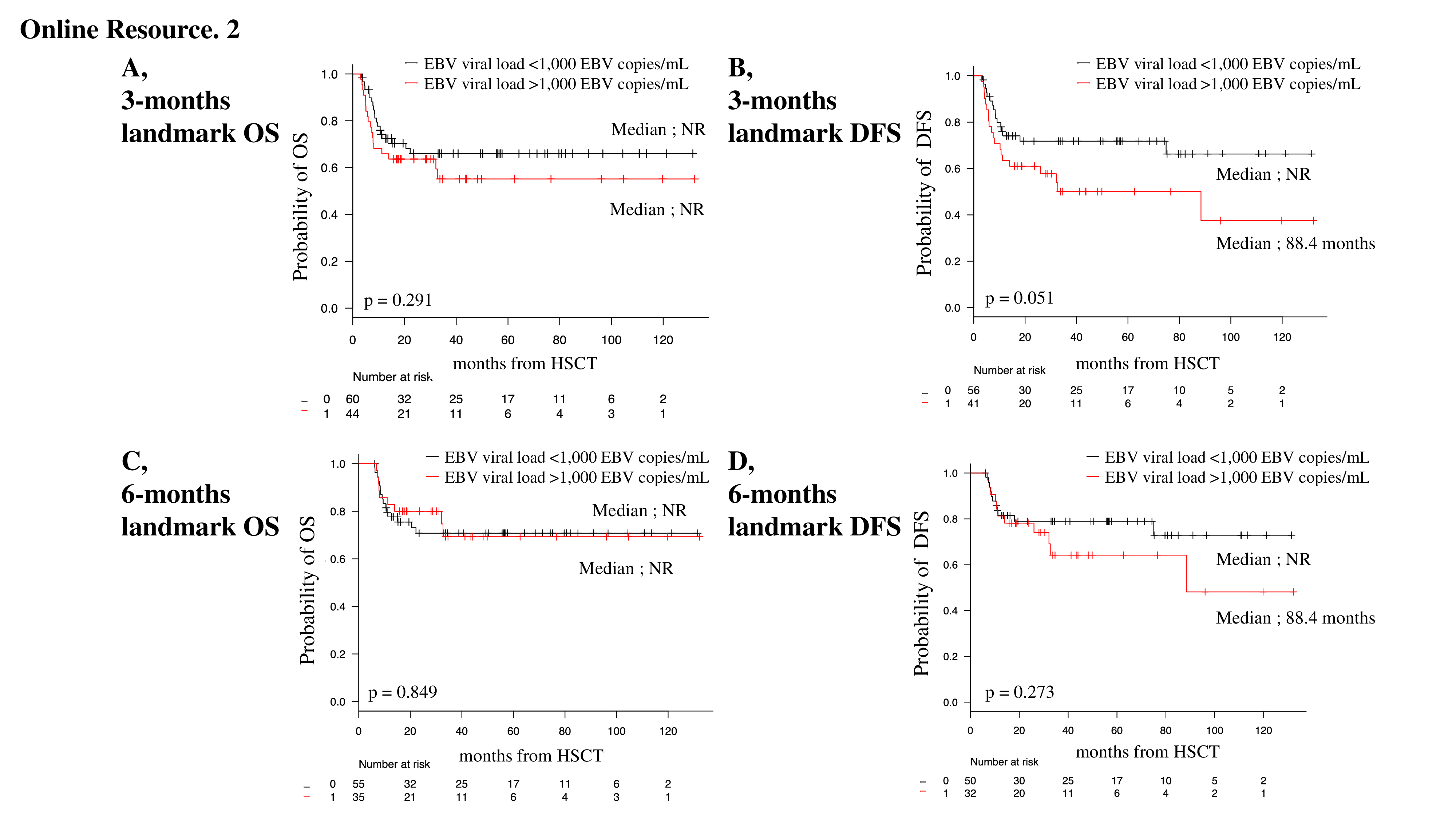

Supplement: Supplementary file 2 — Supplementary file2 (TIFF 1376 KB) [file 277_2023_5596_MOESM2_ESM.tiff]

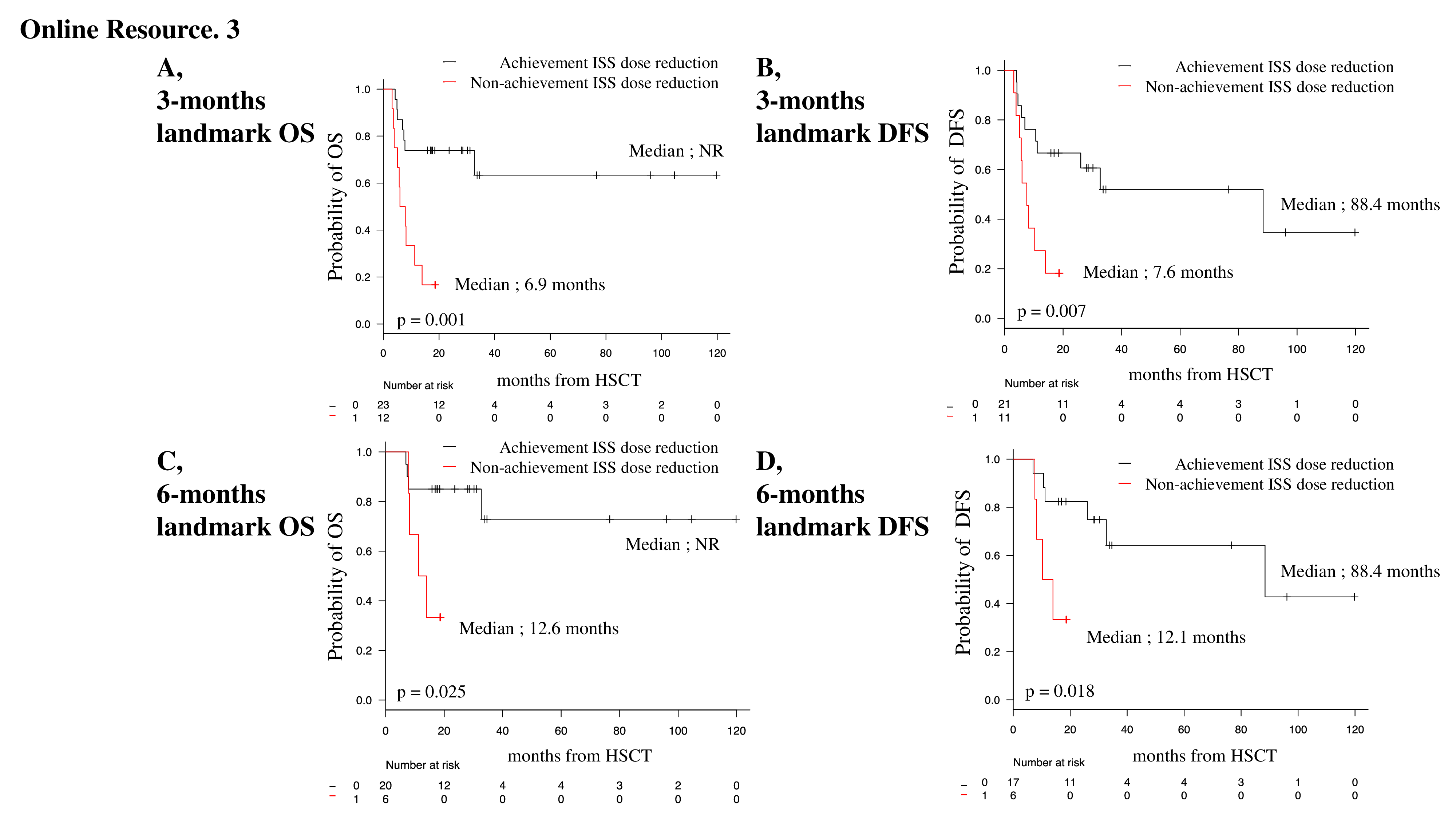

Supplement: Supplementary file 3 — Supplementary file3 (TIFF 1378 KB) [file 277_2023_5596_MOESM3_ESM.tiff]
